# Foreword Special Issue Genomic Instability in Tumor Evolution and Therapy Response

**DOI:** 10.3390/cancers15123080

**Published:** 2023-06-07

**Authors:** Jone Mitxelena, Ana M. Zubiaga

**Affiliations:** 1 Department of Genetics, Physical Anthropology and Animal Physiology, University of the Basque Country, UPV/EHU, 48080 Bilbao, Spain; 2 Ikerbasque, Basque Foundation for Science, 48009 Bilbao, Spain

From an evolutionary perspective, mutations in the DNA molecule act as a source of genetic variation and thus, are beneficial to the adaptation and survival of the species. On the contrary, at the individual level, DNA mutations can have catastrophic effects on our health. Elevated mutation rates increase the likelihood of acquiring variants that favor tumor development and promote the acquisition of aggressive traits in cancer cells [1]. To combat this, cells have developed sophisticated surveillance mechanisms that ensure the integrity of our genomes [2]. These molecular pathways are constantly working to detect and repair any changes in the genome, and to ensure that any new mutations that are produced in the DNA molecule are not transmitted to the next generation of cells. The loss of genome integrity is considered a hallmark of cancer cells; it is widely accepted nowadays that it arises either from defective DNA repair mechanisms or as a consequence of cancer cell-intrinsic anarchic cellular processes, such as unscheduled DNA replication, that overwhelm high-fidelity DNA repair. This Special Issue of *Cancers* features some of the mechanisms that contribute to genomic instability in cancer cells, and describes novel therapeutic approaches targeting this cancer cell hallmark.

DNA damage takes place in the context of chromatin, and thus, chromatin regulation is considered an essential aspect for DNA repair processes. Similarly, mutations have been described in several epigenetic regulators with roles in the caretaking of the human genome. Loss-of-function mutations in the ATRX epigenetic regulator occur in a variety of cancer types, highlighting its oncosuppressive role. ATRX is frequently mutated in glioblastoma (GBM), the most common malignant type of glioma in adults. The overall survival rate of GBM patients is very low, which underscores the urgency for the development of effective strategies for GBM treatment. The group led by Dr. López-Contreras has now identified drugs that exhibit synthetic lethality with ATRX loss and thus, exploit ATRX deficiency for the treatment of GBM patients harboring inactivating mutations in this epigenetic regulator [3]. Through a series of screens and functional assays, authors found that FDA-approved multi-targeted receptor tyrosine kinase (RTK) and platelet-derived growth factor receptor (PDGFR) inhibitors are particularly toxic to ATRX-deficient high-grade glioma cells. The authors further show that high-grade glioma cells lacking ATRX exhibit considerable toxicity when RTKi and temozolomide (TMZ), the current standard treatment for GBM patients, are combined. Altogether, the authors propose that TMZ and RTKi combined therapies may be a valuable strategy to treat high-grade gliomas harboring ATRX mutations.

With the exception of ATRX and a few other examples, efforts to identify mutations in genome caretakers in sporadic (non-hereditary) cancers have had limited success. As a consequence, our knowledge of the molecular basis underlying genome instability in sporadic cancers is quite scarce. The high rate of genomic instability presented by cancer cells may be the result of multi-step failures, ranging from replication fidelity issues, incorrect distribution of chromosomes, chromothripsis and telomere dysfunction. Loss of genome integrity has been mainly attributed to the stress that overwhelms high-fidelity DNA replication, known as replication stress. Indeed, it is widely accepted that the onset of replication stress represents an early event during tumorigenesis [4]. Dr. Zubiaga and collaborators now propose that the transcriptional program driven by E2F transcription factors is key to coping with the exceptionally high levels of basal or drug-induced DNA replication stress in prostate cancer cells [5]. E2F1 and E2F2 factors are highly expressed in several cancer types, including prostate cancer (PCa), and their overexpression often correlates with poor clinical outcomes. In prostate cancer cells, the inhibition of E2F activity induces S-phase specific DNA damage, sensitizes prostate cancer cells to drug-induced replication stress and cellular toxicity and undermines tumorigenic capacity of PCa cells in xenograft models. Mechanistically, E2F activity abrogates inappropriate activation of the mitotic kinase CDK1 during the S phase, which is key to ensuring genome stability and viability of prostate cancer cells. Unscheduled activation of CDK1 through the cell cycle is toxic for mammalian cells, which underscores the importance of identifying mechanisms that constrain CDK1 action out of mitosis [6,7]. In this issue, Dr. Zubiaga and coworkers unveil that E2F activity is critical for limiting CDK1 activity in S-phase, and for preventing the ensuing replication stress and catastrophic DNA damage. From a therapeutic perspective, this work highlights the suitability of targeting E2F for the treatment of prostate cancer in combination with the current standard treatments.

Combinatorial chemotherapy regimens are the mainstay options for the treatment of several malignancies. However, cancer cells often evade the resulting genotoxic stress by activating resistance mechanisms. In an effort to improve treatment efficacy, a great deal of effort is being invested into identifying the mechanisms of chemotherapy resistance that cancer cells exhibit. Dr. Shao and collaborators have now identified a specific phosphorylation in a DNA repair regulator named VCP as a potential predictive biomarker of chemo-resistance in pancreatic ductal adenocarcinoma (PDAC) [8]. By regulating chromatin-associated protein homeostasis and reorganization, VCP ATPase is involved in the regulation of virtually all DNA transacting processes including DNA replication, transcription and DNA repair [9]. Currently, a VCP inhibitor is being evaluated in Phase I clinical trials for acute myeloid leukemia and myelodysplastic syndromes treatment, and it is also expected to undergo clinical testing for solid tumors. According to the work of Dr. Shao and coworkers in this issue, DNA damage-induced VCP phosphorylation in Ser784 is essential for DNA repair in PDAC cells, and the intratumoral levels of this specific phosphorylation predict both the response to chemotherapy and the chemosensitizing effect of pharmacological VCP inhibitors. Thus, VCP might be considered a potential sensitizing target for combinatorial chemotherapy treatments in PDAC patients.

There is mounting evidence suggesting that genomic instability facilitates immune system detection of cancer cells. For instance, neo-antigens produced by point mutations or genomic rearrangements are presented to immune cells to facilitate their recognition and extinction. Moreover, immune cells are also activated by inflammatory responses boosted in response to DNA damage accumulation in cancer cells. Thus, clearance by the immune system is a significant hurdle that tumor cells must overcome during carcinogenesis. To escape immune control, cancer cells exploit a variety of molecular strategies that block the recognition and attack inflicted by the immune system [10]. Flawed antigen presentation due to the altered expression of the human leucocyte antigen (HLA) molecules on the tumor cell surface is a common mechanism utilized by cancer cells to evade the immune system. Moreover, the altered expression of tumor HLA has been proposed to be an escape strategy against immunotherapy, which is linked to reduced T-cell cytotoxicity. Altered HLA expression in tumors is attributed to multiple molecular mechanisms, including mutations on HLA and β2-microglobulin (B2M) genes, transcriptional downregulation, HLA allelic losses, hypermethylation and loss of heterozygosity (LOH) involving HLA genes. In this issue, Dr. Aptsiauri and coworkers investigate the incidence and the mechanism of loss of heterozygosity at chromosomes 6 and 15 (LOH-6 and LOH-15), where HLA class I genes are located [11]. Single Nucleotide Polymorphism (SNP) analyses employing SNP-arrays in several human tumor cell lines and cancer samples of different histological types demonstrate the prevalence of loss of heterozygosity involving HLA-I and B2M genes. Understanding the immune evasion mechanisms exploited by cancer cells might be of value to discovering biomarkers that can predict tumor evolution and the response to immunotherapy.

With the development of new and more effective anti-tumor therapies, patient management based on novel biomarkers and population-based cancer screening programs, the survival and overall quality of life of cancer patients have improved considerably in recent years. However, although treatment protocols are being updated continuously to increase survival rates while diminishing therapy-related toxicity, patients frequently experience important adverse side effects seriously affecting their quality of life during treatment and afterwards throughout their lives. A review article by Dr. Vermeij in this issue analyzes, in detail, major clinical side effects in cancer patients and survivors, and discusses potential underlying mechanisms that might cause these toxicities [12]. This review article focuses on the toxicity induced by radiation therapy and by broadly used chemotherapy, and considers how the nature of DNA lesions induced by the treatment, together with drug-intrinsic properties, such as biodistribution, influence the type and the degree of cancer therapy-induced toxicity. Considering that both the cure rate for cancer and the overall life expectancy has increased over the last few decades, a better understanding of therapy-induced side effects might facilitate the implementation of beneficial interventions to improve the quality of life of cancer patients and survivors.
